# Survival in Patients With Metastatic Prostate Cancer Undergoing Radiotherapy: The Importance of Prostate-Specific Antigen-Based Stratification

**DOI:** 10.3389/fonc.2021.706236

**Published:** 2021-06-10

**Authors:** Zijian Tian, Lingfeng Meng, Xin Wang, Xuan Wang, Tianming Ma, Miao Wang, Qiuzi Zhong, Yaqun Zhang, Ming Liu

**Affiliations:** ^1^ Department of Urology, Beijing Hospital, National Center of Gerontology, Institute of Geriatric Medicine, Chinese Academy of Medical Sciences, Beijing, China; ^2^ Graduate School of Peking Union Medical College, Chinese Academy of Medical Sciences, Beijing, China

**Keywords:** prostate-specific antigen, metastatic prostate cancer, SEER, survival analysis, radiotherapy

## Abstract

**Objectives:**

To explore the effectiveness of radiotherapy in mPCa patients with different PSA stratifications based on the cancer database of a large population.

**Background:**

Screening criteria for patients with metastatic prostate cancer, who are candidates for radiotherapy, are rarely reported.

**Patients and Methods:**

We identified 22,604 patients with metastatic prostate cancer in the Surveillance, Epidemiology, and End Results database and divided them into a radiotherapy group and a control group. Patients with metastatic prostate cancer were divided into subgroups according to their levels of prostate-specific antigen to evaluate the efficacy of radiotherapy. They were also divided into six subgroups according to their prostate-specific antigen levels. We used multivariate Cox analysis to evaluate overall survival and cancer-specific survival. After 1:1 propensity score matching, Kaplan-Meier analysis was used to explore the difference in overall survival and cancer-specific survival in the radiotherapy and control group.

**Results:**

In all, 5,505 patients received radiotherapy, compared to 17,099 in the control group. In the multivariate Cox analysis, radiotherapy improved overall survival (hazard ratio [HR]: 0.730, 95% confidence interval [CI]: 0.636–0.838; P<0.001) and cancer-specific survival (HR: 0.764, 95% CI: 0.647–0.903; P=0.002) in patients with a PSA level of 4–10 ng/mL. Similar results were obtained by Kaplan-Meier analysis after 1:1 propensity score matching. In patients with prostate-specific antigen levels between 4–10 ng/mL, the overall survival (P<0.001) and cancer-specific survival (P<0.05) in the radiotherapy group was significantly better than those in the control group.

**Conclusion:**

The result of this large population-based study shows that rigorous selection of appropriate metastatic prostate cancer patients for radiotherapy can benefit prognosis significantly. This can be the basis for future prospective trials.

## Introduction

The incidence of prostate cancer (PCa) has been increasing annually, ranking first among male malignant tumors in the United States. It is estimated that there were 191,930 new cases of PCa in 2020, with 33,330 patients dying from PCa (1). PCa can present in different stages, and metastasis is an important stage adversely affecting prognosis (2, 3). Although the widespread use of prostate-specific antigen (PSA) testing has reduced the incidence of metastatic prostate cancer (mPCa) (4), 6% of PCa patients in the United States and 15.8% in the Netherlands still have metastatic disease at diagnosis (3, 5). The incidence can be as high as 50–64% in Asian countries (6, 7).

Two recent, prominent randomized controlled trials, HORRAD and STAMPEDE, have shown that radiotherapy has no survival benefit in overall unscreened cohorts of mPCa. In the HORRAD trial, 432 mPCa patients with PSA > 20 ng/mL were divided into a radiotherapy and a control group. The results revealed that radiotherapy did not significantly prolong the overall survival (OS) of patients with mPCa [hazard ratio (HR), 0.90; 95% confidence interval (CI): 0.70–1.14; P=0.4] (8). In the STAMPEDE trial of 2,061 mPCa patients with PSA > 30 ng/mL, there were no survival benefits of radiotherapy in the general population (HR: 0.92, 95% CI 0.80–1.06; P=0.27) (9). This was similar to the HORRAD results. However, in the subgroup analysis based on metastatic burden, radiotherapy was found to prolong the OS of patients with oligometastatic PCa. A meta-analysis combining data from the HORRAD and STAMPEDE studies revealed that radiotherapy could increase the absolute value of 3-year OS by 7% if the number of metastases was less than 4 (10). At present, radiotherapy for oligometastatic PCa has become a routine recommendation (11). Patient selection is important for radiotherapy, with clinicians trying to define further clinical parameters for optimal patient selection. However, no study has reported the efficacy of radiotherapy in patients with mPCa under different PSA subgroups.

PSA levels reflect the load of tumor cells. PCa with a higher PSA level is more aggressive and carries a higher risk of death due to cancer. However, recent studies have reported that PSA levels in patients with mPCa are not linearly related to prognosis. Patients with lower PSA levels (≤ 4 ng/mL) exhibited poorer prognoses than patients with higher PSA levels (12). The HORRAD and STAMPEDE studies also included only mPCa patients with PSA levels greater than 20 and 30 ng/mL, respectively. Previous retrospective studies (13–15) did not stratify mPCa patients according to PSA levels to explore the efficacy of radiotherapy. Therefore, the purpose of this study was to explore the effectiveness of radiotherapy in mPCa patients with different PSA stratifications based on the cancer database of a large population.

## Materials and Methods

### Study Population

The Surveillance, Epidemiology, and End Results (SEER) database is the authoritative cancer statistics database in the United States. It covers about 28% of the clinical cancer patients in the United States and records the morbidity, mortality, and illness of these patients among other clinical information. In the SEER database (2004–2015), we identified 22,604 mPCa patients who met our inclusion criteria. The inclusion criteria were as follows: pathological diagnosis of prostate cancer, metastatic disease at the time of diagnosis, age > 18 years, and having received radiotherapy. The exclusion criteria were as follows: those who underwent radical prostatectomy, patients whose radiotherapy details were incomplete, patients whose PSA information was unrecorded, and patients whose survival time information was lacking. Age, race, Gleason score, T stage, N stage, M stage, and PSA level were recorded for patients who met the criteria.

### Statistical Analysis

This study’s main outcome index is OS; the secondary outcome index is cancer-specific survival (CSS). Pearson’s chi-square analysis was used to determine the variables between different treatment groups. We stratified the data according to PSA levels (< 4.0, 4.1–10.0, 10.1–20.0, 20.1–40.0, 40.1–80.0, > 80.1 ng/mL). Covariates were included in univariate and multivariate Cox regression analyses to determine the prognostic HR and 95% CI of the treatment group. In the multivariate analysis, PSA (4.1–10.0 ng/mL) was used as a reference to explore the relative HR of other subgroups. To balance covariance and reduce deviation in the evaluation of therapeutic effect, we measured OS and CSS using the Kaplan-Meier (KM) method at 1:1 propensity score matching in the radiotherapy group and control group of each PSA subgroup. We designed the analysis for two-sided tests using IBM SPSS Statistics for Windows, Version 25.0. (IBM Corp., Armonk, NY). Statistical significance was set at P < 0.05.

## Results

### Demographics and Pathological Characteristics

A total of 22,604 mPCa patients who met the inclusion criteria were identified from the SEER database. There were 5,505 patients in the radiotherapy group and 17,099 participants in the control group. Patient characteristics are shown in **Table 1**.

**Table 1:** Clinicopathological characteristics of the cohort by treatment groups.

Characteristics	Radiotherapy	Control	P value^a^
	(n=5505; %)	(n=17099; %)	
**Age**			<0.001
≤65	2303 (41.8%)	5564 (32.5%)	
>65	3202 (58.2%)	11535 (67.5%)	
**Race**			0.672
Caucasians	4106 (74.6%)	12761 (74.6%)	
African Americans	1044 (19.0%)	3185 (18.6%)	
Other/unknown	355 (6.4%)	1153 (6.7%)	
**Gleason**			<0.001
≤6	193 (3.5%)	501 (2.9%)	
7	750 (13.6%)	2384 (13.9%)	
8-10	3092 (56.2%)	10385 (60.7%)	
Unknown	1470 (26.7%)	3829 (22.4%)	
**T**			0.006
≤T1	1334 (24.2%)	4305 (25.2%)	
T2	1662 (30.2%)	5480 (32.0%)	
T3	569 (10.3%)	1610 (9.4%)	
T4	749 (13.6%)	2154 (12.6%)	
Tx	1191 (21.6%)	3550 (20.8%)	
**N**			<0.001
N0	2959 (53.8%)	8901 (52.1%)	
N1	1396 (25.4%)	4128 (24.1%)	
Nx	1150 (20.9%)	4070 (23.8%)	
**M**			<0.001
M1a	228 (4.1%)	1043 (6.1%)	
M1b	3996 (72.6%)	12256 (71.7%)	
M1c	1142 (20.7%)	3275 (19.2%)	
M1x	139 (2.5%)	525 (3.1%)	
**PSA**			<0.001
<4 ng/ml	154 (2.8%)	403 (2.4%)	
4.1-10 ng/ml	551 (10.0%)	1146 (6.7%)	
10.1-20 ng/ml	596 (10.8%)	1645 (9.6%)	
20.1-40 ng/ml	622 (11.3%)	2019 (11.8%)	
40.1-80 ng/ml	666 (12.1%)	2134 (12.5%)	
>80.1 ng/ml	2916 (53.0%)	9752 (57.0%)	

Data are present as n (%).

PSA, prostate-specific antigen.

^a^Chi-square test as appropriate.

### Statistical Analysis

Univariate and multivariate analysis were used to determine whether there was a statistical correlation between the PSA subgroups and the prognosis of patients within each subgroup. With each PSA subgroup, we included age, race, Gleason score, treatment groups, T stage, N stage, and M stage in a univariate Cox analysis. The univariate Cox analysis showed that the PSA group with the highest OS was the PSA 4.1–10.0 ng/mL subgroup (P < 0.001), while the PSA groups correlated with CSS were the PSA 4.1–10.0 ng/mL and PSA > 80.1 ng/mL subgroups (P<0.05) (**Supplementary Table 1**). All significant factors in the univariate Cox analysis (e.g., 4.1–10.0 ng/mL, 40.1–80.0 ng/mL, and > 80.1 ng/mL subgroups) were included in the multivariate Cox regression analysis. The results showed that radiotherapy could significantly improve OS (HR: 0.730, 95% CI: 0.636–0.838; P < 0.001) and CSS (HR: 0.764,95% CI: 0.647–0.903; P=0.002) of mPCa in the PSA 4.1–10.0 ng/mL subgroup (**Table 2**). In the PSA > 80.1 ng/mL subgroup, the radiotherapy group was associated with worse CSS (HR: 1.065, 95% CI: 1.009–1.124; P=0.023) (**Supplementary Table 2**).

**Table 2:** Multivariate Cox regression analysis of treatment groups in PSA 4.1-10ng/ml subgroup.

Clinicopathological variables	OS multivariate analysis		CSS multivariate analysis	
	HR (95%CI)	P value	HR (95%CI)	P value
**Group**				
Control group	Reference		Reference	
Radiotherapy group	0.730 (0.636-0.838)	<0.001	0.764 (0.647-0.903)	0.002
**Age at diagnosis**				
≤65	Reference		/	/
>65	1.129 (0.983-1.297)	0.086	/	/
**Race**				
Caucasians	Reference		Reference	
African Americans	0.767 (0.620-0.948)	0.014	0.710 (0.541-0.930)	0.013
Other/Unknown	0.704 (0.535-0.927)	0.013	0.678 (0.485-0.948)	0.023
**T**				
≤T1	Reference		Reference	
T2	1.009 (0.867-1.174)	0.906	0.900 (0.747-1.085)	0.268
T3	0.845 (0.669-1.069)	0.161	0.862 (0.658-1.129)	0.282
T4	1.677 (1.350-2.083)	<0.001	1.760 (1.374-2.255)	<0.001
Tx	1.348 (1.035-1.756)	0.027	1.265 (0.915-1.750)	0.155
**N**				
N0	Reference		Reference	
N1	1.267 (1.065-1.508)	0.008	1.404 (1.151-1.714)	0.001
Nx	0.692 (1.036-0.871)	0.692	1.079 (0.873-1.335)	0481
**M**				
M1a	Reference		Reference	
M1b	1.304 (0.991-1.716)	0.058	1.634 (1.154-2.315)	0.006
M1c	1.636 (1.220-2.195)	0.001	2.136 (1.477-3.089)	<0.001
M1x	1.936 (1.269-2.953)	0.002	2.229 (1.311-3.793)	0.003
**Gleason**				
≤6	Reference		Reference	
7	1.673 (1.258-2.225)	<0.001	2.701 (1.692-4.311)	<0.001
8-10	2.619 (2.021-3.395)	<0.001	5.764 (3.735-8.894)	<0.001
Unknown	3.526 (2.548-4.880)	<0.001	6.263 (3.802-10.316)	<0.001

HR, hazard ratio; 95%CI, 95% confidence intervals; OS, overall survival; CSS, cancer specific survival.

**Figure 1:**
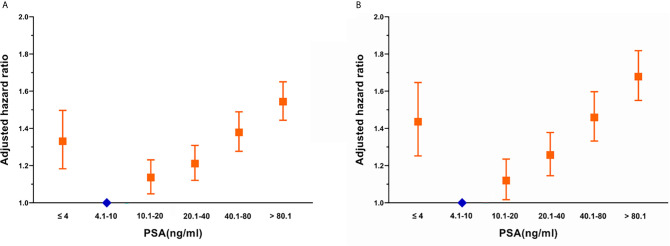
Adjusted hazard ratios with 95% confidence intervals for the association between PSA and **(A)** overall survival and **(B)** cancer-specific survival.

In the multivariate analysis, using the PSA 4.1–10.0 ng/mL subgroup as the reference, the distribution of OS in the SEER cohort showed a U-shaped distribution relative to PSA (**Figure 1**), the HRs of the PSA < 4.0, 10.1–20.0, 20.1–40.0, 40.1–80.0, and > 80.1 ng/mL groups were 1.331, 1.136, 1.211, 1.379, and 1.544, respectively (**Supplementary Table 3**). When CSS was used as the outcome index, the HRs of the PSA < 4.0, 10.1–20.0, 20.1–40.0, 40.1–80.0, and > 80.1 ng/mL groups were 1.436, 1.120, 1.257, 1.459, and 1.679, respectively (**Supplementary Table 3**). CSS and PSA showed a similar U-shaped distribution (**Figure 1**).

### 1:1 Propensity Score Matching

**Figure 2:**
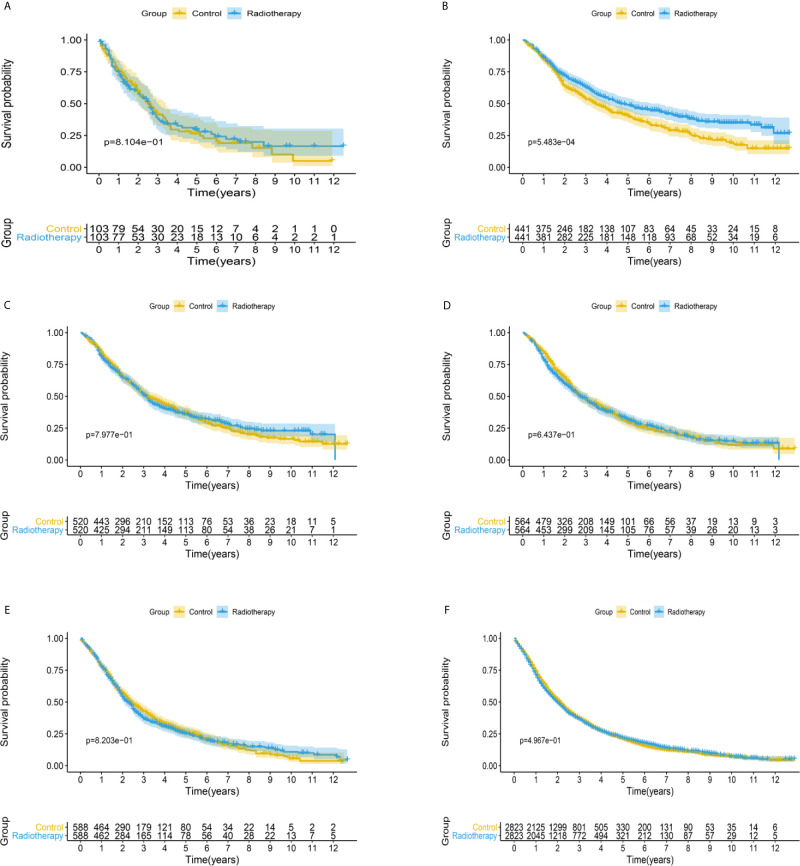
Kaplan-Meier survival analysis of overall survival in **(A)** PSA <4 ng/mL, **(B)** PSA 4.1–10 ng/mL, **(C)** PSA 10.1–20 ng/mL, **(D)** PSA 20.1–40 ng/mL, **(E)** PSA 40.1–80 ng/mL, and **(F)** PSA >80.1 ng/mL subgroups.

**Figure 3:**
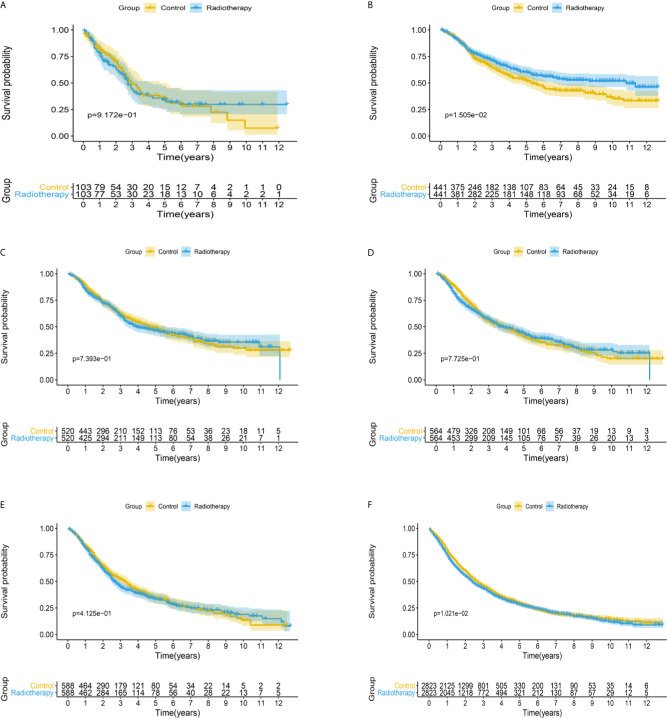
Kaplan-Meier survival analysis of cancer-specific survival in **(A)** PSA <4 ng/mL, **(B)** PSA 4.1–10 ng/mL, **(C)** PSA 10.1–20 ng/mL, **(D)** PSA 20.1–40 ng/mL, **(E)** PSA 40.1–80 ng/mL, and **(F)** PSA >80.1 ng/mL subgroups.

After matching with a 1:1 tendency score, there was no significant difference in age, race, Gleason score, T stage, N stage, and M stage between the radiotherapy and control group in each PSA subgroup (**Tables 3**–**8**). KM analysis showed that radiotherapy could significantly improve OS only in patients of the PSA 4.1–10.0 ng/mL subgroup (P < 0.001). There was no significant difference in OS between the radiotherapy and control groups in other PSA subgroups (**Figure 2**). When CSS was used as the outcome index, KM analysis showed that radiotherapy was most beneficial in mPCa patients with PSA levels of 4.1–10.0 ng/mL. In the PSA > 80.1 ng/mL subgroup, the CSS of the radiotherapy group was inferior to that of the control group (P < 0.05), but there was no significant difference in the improvement of prognosis among other PSA subgroups (**Figure 3**).

**Table 3:** Clinicopathological characteristics of the PSA <4 ng/ml subgroup stratified according to treatment modality with and without propensity score matching.

Characteristics	Radiotherapy	Control	P value^a^	Propensity score adjusted radiotherapy	Propensity score adjusted control	P value^b^
	(n=154)	(n=403)		(n=103)	(n=103)	
**Age**			0.240			0.876
≤65	55 (35.7%)	123 (30.5%)		29 (28.2%)	28 (27.2%)	
>65	99 (64.3%)	280 (69.5%)		74 (71.8%)	75 (72.8%)	
**Race**			0.632			1.000
Caucasians	128 (83.1%)	337 (83.6%)		95 (92.2%)	95 (92.2%)	
African Americans	21 (13.6%)	47 (11.7%)		6 (5.8%)	6 (5.8%)	
Other/unknown	5 (3.2%)	19 (4.7%)		2 (1.9%)	2 (1.9%)	
**Gleason**			0.003			0.953
≤6	8 (5.2%)	20 (5.0%)		2 (1.9%)	2 (1.9%)	
7	29 (18.8%)	34 (8.4%)		9 (8.7%)	7 (6.8%)	
8-10	85 (55.2%)	274 (68.0%)		69 (67.0%)	72 (69.9%)	
Unknown	32 (20.8%)	75 (18.6%)		23 (22.3%)	22 (21.4%)	
**T**			0.014			1.000
≤T1	27 (17.5%)	107 (26.6%)		20 (19.4%)	19 (18.4%)	
T2	54 (35.1%)	144 (35.7%)		34 (33.0%)	34 (33.0%)	
T3	22 (14.3%)	30 (7.4%)		13 (12.6%)	13 (12.6%)	
T4	32 (20.8%)	60 (14.9%)		21 (20.4%)	21 (20.4%)	
Tx	19 (12.3%)	62 (15.4%)		15 (14.6%)	16 (15.5%)	
**N**			0.065			1.000
N0	101 (65.6%)	229 (56.8%)		72 (69.9%)	72 (69.9%)	
N1	25 (16.2%)	102 (25.3%)		15 (14.6%)	15 (14.6%)	
Nx	28 (18.2%)	72 (17.9%)		16 (15.5%)	16 (15.5%)	
**M**			0.374			0.784
M1a	8 (5.2%)	39 (9.7%)		4 (3.9%)	7 (6.8%)	
M1b	95 (61.7%)	243 (60.3%)		70 (68.0%)	65 (63.1%)	
M1c	43 (27.9%)	104 (25.8%)		28 (27.2%)	30 (29.1%)	
M1x	8 (5.2%)	17 (4.2%)		1 (1.0%)	1 (1.0%)	

Data are present as n (%).

^a^Comparing radiotherapy versus control group (unmatched).

^b^Comparing radiotherapy versus control group (propensity score-adjusted cohorts).

**Table 4:** Clinicopathological characteristics of the PSA 4.1-10 ng/ml subgroup stratified according to treatment modality with and without propensity score matching.

Characteristics	Radiotherapy	Control	P value^a^	Propensity score adjusted radiotherapy	Propensity score adjusted control	P value^b^
	(n=551)	(n=1146)		(n=441)	(n=441)	
**Age**			0.210			0.822
≤65	188 (34.1%)	328 (28.6%)		124 (28.1%)	121 (27.4%)	
>65	363 (65.9%)	818 (71.4%)		317 (71.9%)	320 (72.6%)	
**Race**			0.663			0.967
Caucasians	445 (80.8%)	930 (81.2%)		372 (84.4%)	373 (84.6%)	
African Americans	73 (13.2%)	138 (12.0%)		45 (10.2%)	43 (9.8%)	
Other/unknown	33 (6.0%)	78 (6.8%)		24 (5.4%)	25 (5.7%)	
**Gleason**			<0.001			0.997
≤6	74 (13.4%)	104 (9.1%)		45 (10.2%)	44 (10.0%)	
7	128 (23.2%)	193 (16.8%)		90 (20.4%)	92 (20.9%)	
8-10	304 (55.2%)	730 (63.7%)		277 (62.8%)	277 (62.8%)	
Unknown	45 (8.2%)	119 (10.4%)		29 (6.6%)	28 (6.3%)	
**T**			0.006			0.993
≤T1	227 (41.2%)	391 (34.1%)		178 (40.4%)	179 (40.6%)	
T2	185 (33.6%)	413 (36.0%)		162 (36.7%)	162 (36.7%)	
T3	49 (8.9%)	117 (10.2%)		37 (8.4%)	35 (7.9%)	
T4	59 (10.7%)	112 (9.8%)		41 (9.3%)	44 (10.0%)	
Tx	31 (5.6%)	113 (9.9%)		23 (5.2%)	21 (4.8%)	
**N**			0.077			0.988
N0	386 (70.1%)	741 (64.7%)		322 (73.0%)	324 (73.5%)	
N1	90 (16.3%)	211 (18.4%)		69 (15.6%)	68 (15.4%)	
Nx	75 (13.6%)	194 (16.9%)		50 (11.3%)	49 (11.1%)	
**M**			0.350			0.939
M1a	29 (5.3%)	76 (6.6%)		19 (4.3%)	19 (4.3%)	
M1b	401 (72.8%)	814 (71.0%)		352 (79.8%)	350 (79.4%)	
M1c	109 (19.8%)	217 (18.9%)		66 (15.0%)	66 (15.0%)	
M1x	12 (2.2%)	39 (3.4%)		4 (0.9%)	6 (1.4%)	

Data are present as n (%).

^a^Comparing radiotherapy versus control group (unmatched).

^b^Comparing radiotherapy versus control group (propensity score-adjusted cohorts).

**Table 5:** Clinicopathological characteristics of the PSA 10.1-20 ng/ml subgroup stratified according to treatment modality with and without propensity score matching.

Characteristics	Radiotherapy	Control	P value^a^	Propensity score adjusted radiotherapy	Propensity score adjusted control	P value^b^
	(n=596)	(n=1645)		(n=520)	(n=520)	
**Age**			0.009			0.894
≤65	202 (33.9%)	463 (28.1%)		164 (31.5%)	162 (31.2%)	
>65	394 (66.1%)	1182 (71.9%)		356 (68.5%)	358 (68.8%)	
**Race**			0.407			0.651
Caucasians	477 (80.0%)	1300 (79.0%)		434 (83.5%)	440 (84.6%)	
African Americans	88 (14.8%)	234 (14.2%)		61 (11.7%)	61 (11.7%)	
Other/unknown	31 (5.2%)	111 (6.7%)		25 (4.8%)	19 (3.7%)	
**Gleason**			0.558			0.979
≤6	36 (6.0%)	111 (6.7%)		26 (5.0%)	27 (5.2%)	
7	113 (19.0%)	276 (16.8%)		96 (18.5%)	101 (19.4%)	
8-10	371 (62.2%)	1027 (62.4%)		340 (65.4%)	335 (64.4%)	
Unknown	76 (12.8%)	231 (14.0%)		58 (11.2%)	57 (11.0%)	
**T**			0.066			0.999
≤T1	198 (33.2%)	536 (32.6%)		177 (34.0%)	174 (33.5%)	
T2	203 (34.1%)	571 (34.7%)		188 (36.2%)	189 (36.3%)	
T3	68 (11.4%)	139 (8.4%)		47 (9.0%)	46 (8.8%)	
T4	71 (11.9%)	187 (11.4%)		62 (11.9%)	63 (12.1%)	
Tx	56 (9.4%)	212 (12.9%)		46 (8.8%)	48 (9.2%)	
**N**			<0.001			1.000
N0	410 (68.8%)	1001 (60.9%)		378 (72.7%)	378 (72.7%)	
N1	118 (19.8%)	311 (18.9%)		84 (16.2%)	84 (16.2%)	
Nx	68 (11.4%)	333 (20.2%)		58 (11.2%)	58 (11.2%)	
**M**			0.453			0.895
M1a	33 (5.5%)	118 (7.2%)		15 (2.9%)	19 (3.7%)	
M1b	430 (72.1%)	1192 (72.5%)		412 (79.2%)	411 (79.0%)	
M1c	118 (19.8%)	294 (17.9%)		88 (16.9%)	86 (16.5%)	
M1x	15 (2.5%)	41 (2.5%)		5 (1.0%)	4 (0.8%)	

Data are present as n (%).

^a^Comparing radiotherapy versus control group (unmatched).

^b^Comparing radiotherapy versus control group (propensity score-adjusted cohorts).

**Table 6:** Clinicopathological characteristics of the PSA 20.1-40 ng/ml subgroup stratified according to treatment modality with and without propensity score matching.

Characteristics	Radiotherapy	Control	P value^a^	Propensity score adjusted radiotherapy	Propensity score adjusted control	P value^b^
	(n=622)	(n=2019)		(n=564)	(n=564)	
**Age**			0.001			0.850
≤65	224 (36.0%)	587 (29.1%)		186 (33.0%)	189 (33.5%)	
>65	398 (64.0%)	1432 (70.9%)		378 (67.0%)	375 (66.5%)	
**Race**			0.552			0.991
Caucasians	491 (78.9%)	1623 (80.4%)		467 (82.8%)	466 (82.6%)	
African Americans	89 (14.3%)	255 (12.6%)		68 (12.1%)	68 (12.1%)	
Other/unknown	42 (6.8%)	141 (7.0%)		29 (5.1%)	30 (5.3%)	
**Gleason**			0.024			0.919
≤6	24 (3.9%)	62 (3.1%)		14 (2.5%)	17 (3.0%)	
7	91 (14.6%)	362 (17.9%)		83 (14.7%)	84 (14.9%)	
8-10	396 (63.7%)	1317 (65.2%)		372 (66.0%)	374 (66.3%)	
Unknown	111 (17.8%)	278 (13.8%)		95 (16.8%)	89 (15.8%)	
**T**			0.083			0.906
≤T1	167 (26.8%)	600 (29.7%)		158 (28.0%)	160 (28.4%)	
T2	204 (32.8%)	705 (34.9%)		186 (33.0%)	181 (32.1%)	
T3	78 (12.5%)	230 (11.4%)		69 (12.2%)	77 (13.7%)	
T4	64 (10.3%)	214 (10.6%)		54 (9.6%)	47 (8.3%)	
Tx	109 (17.5%)	270 (13.4%)		97 (17.2%)	99 (17.6%)	
**N**			0.704			0.869
N0	367 (59.0%)	1213 (60.1%)		345 (61.2%)	344 (61.0%)	
N1	126 (20.3%)	418 (20.7%)		113 (20.0%)	108 (19.1%)	
Nx	129 (20.7%)	388 (19.2%)		106 (18.8%)	112 (19.9%)	
**M**			0.905			0.642
M1a	40 (6.4%)	146 (7.2%)		33 (5.9%)	32 (5.7%)	
M1b	452 (72.7%)	1449 (71.8%)		427 (75.7%)	414 (73.4%)	
M1c	111 (17.8%)	366 (18.1%)		92 (16.3%)	108 (19.1%)	
M1x	19 (3.1%)	58 (2.9%)		12 (2.1%)	10 (1.8%)	

Data are present as n (%).

^a^Comparing radiotherapy versus control group (unmatched).

^b^Comparing radiotherapy versus control group (propensity score-adjusted cohorts).

**Table 7:** Clinicopathological characteristics of the PSA 40.1-80ng/ml subgroup stratified according to treatment modality with and without propensity score matching.

Characteristics	Radiotherapy	Control	P value^a^	Propensity score adjusted radiotherapy	Propensity score adjusted control	P value^b^
	(n=666)	(n=2134)		(n=588)	(n=588)	
**Age**			<0.001			0.904
≤65	261 (39.2%)	661 (31.0%)		217 (36.9%)	215 (36.6%)	
>65	405 (60.8%)	1473 (69.0%)		371 (63.1%)	373 (63.4%)	
**Race**			0.160			0.988
Caucasians	511 (76.7%)	1684 (78.9%)		477 (81.1%)	475 (80.8%)	
African Americans	98 (14.7%)	313 (14.7%)		75 (12.8%)	76 (12.9%)	
Other/unknown	57 (8.6%)	137 (6.4%)		36 (6.1%)	37 (6.3%)	
**Gleason**			0.022			1.000
≤6	11 (1.7%)	43 (2.0%)		5 (0.9%)	5 (0.9%)	
7	108 (16.2%)	336 (15.7%)		81 (13.8%)	80 (13.6%)	
8-10	406 (61.0%)	1409 (66.0%)		389 (66.2%)	389 (66.2%)	
Unknown	141 (21.2%)	346 (16.2%)		113 (19.2%)	114 (19.4%)	
**T**			0.404			0.999
≤T1	176 (26.4%)	604 (28.3%)		161 (27.4%)	157 (26.7%)	
T2	195 (29.3%)	677 (31.7%)		178 (30.3%)	179 (30.4%)	
T3	83 (12.5%)	233 (10.9%)		72 (12.2%)	72 (12.2%)	
T4	89 (13.4%)	262 (12.3%)		66 (11.2%)	68 (11.6%)	
Tx	123 (18.5%)	358 (16.8%)		111 (18.9%)	112 (19.0%)	
**N**			0.932			0.975
N0	360 (54.1%)	1157 (54.2%)		326 (55.4%)	325 (55.3%)	
N1	162 (24.3%)	529 (24.8%)		136 (23.1%)	139 (23.6%)	
Nx	144 (21.6%)	448 (21.0%)		126 (21.4%)	124 (21.1%)	
**M**			0.205			0.999
M1a	32 (4.8%)	150 (7.0%)		19 (3.2%)	19 (3.2%)	
M1b	482 (72.4%)	1530 (71.7%)		449 (76.4%)	451 (76.7%)	
M1c	131 (19.7%)	386 (18.1%)		111 (18.9%)	109 (18.5%)	
M1x	21 (3.2%)	68 (3.2%)		9 (1.5%)	9 (1.5%)	

Data are present as n (%).

^a^Comparing radiotherapy versus control group (unmatched).

^b^Comparing radiotherapy versus control group (propensity score-adjusted cohorts).

**Table 8:** Clinicopathological characteristics of the PSA >80.1 ng/ml subgroup stratified according to treatment modality with and without propensity score matching.

Characteristics	Radiotherapy	Control	P value^a^	Propensity score adjusted radiotherapy	Propensity score adjusted control	P value^b^
	(n=2916)	(n=9752)		(n=2823)	(n=2823)	
**Age**			<0.001			0.873
≤65	1373 (47.1%)	3402 (34.9%)		1301 (46.1%)	1307 (46.3%)	
>65	1543 (52.9%)	6350 (65.1%)		1522 (53.9%)	1516 (53.7%)	
**Race**			0.611			0.990
Caucasians	2054 (70.4%)	6887 (70.6%)		2024 (71.7%)	2028 (71.8%)	
African Americans	675 (23.1%)	2198 (22.5%)		636 (22.5%)	634 (22.5%)	
Other/unknown	187 (6.4%)	667 (6.8%)		163 (5.8%)	161 (5.7%)	
**Gleason**			<0.001			0.991
≤6	40 (1.4%)	161 (1.7%)		27 (1.0%)	29 (1.0%)	
7	281 (9.6%)	1183 (12.1%)		267 (9.5%)	271 (9.6%)	
8-10	1530 (52.5%)	5628 (57.7%)		1513 (53.6%)	1511 (53.5%)	
Unknown	1065 (36.5%)	2780 (28.5%)		1016 (36.0%)	1012 (35.8%)	
**T**			<0.001			1.000
≤T1	539 (18.5%)	2067 (21.2%)		518 (18.3%)	518 (18.3%)	
T2	821 (28.2%)	2970 (30.5%)		807 (28.6%)	810 (28.7%)	
T3	269 (9.2%)	861 (8.8%)		252 (8.9%)	251 (8.9%)	
T4	434 (14.9%)	1319 (13.5%)		409 (14.5%)	406 (14.4%)	
Tx	853 (29.3%)	2535 (26.0%)		837 (29.6%)	838 (29.7%)	
**N**			<0.001			1.000
N0	1335 (45.8%)	4560 (46.8%)		1301 (46.1%)	1302 (46.1%)	
N1	875 (30.0%)	2557 (26.2%)		845 (29.9%)	844 (29.9%)	
Nx	706 (24.2%)	2635 (27.0%)		677 (24.0%)	677 (24.0%)	
**M**			<0.001			0.998
M1a	86 (2.9%)	514 (5.3%)		75 (2.7%)	77 (2.7%)	
M1b	2136 (73.3%)	7028 (72.1%)		2103 (74.5%)	2105 (74.6%)	
M1c	630 (21.6%)	1908 (19.6%)		598 (21.2%)	594 (21.0%)	
M1x	64 (2.2%)	302 (3.1%)		47 (1.7%)	47 (1.7%)	

Data are present as n (%).

^a^Comparing radiotherapy versus control group (unmatched).

^b^Comparing radiotherapy versus control group (propensity score-adjusted cohorts).

## Discussion

Large-scale population-based cohort analyses of PCa patients can provide important guidelines for clinical treatment and further prospective studies. At present, a stratified analysis of 22,604 patients with mPCa in the SEER database was conducted to determine whether PSA grouping would affect the efficacy of radiotherapy.

The direct cytotoxic effects of local radiotherapy for PCa have been widely reported (16). The biological mechanism of radiotherapy may be related to tumor cell death and changes in the tumor microenvironment (TME) induced by radiation (17). In short, radiation directly damages the TME, triggering the release of immunostimulatory factors, such as heat shock protein 70, enhancing the anti-tumor activity of immune cells (18, 19). Radiotherapy also changes the vascular endothelial cells of the tumor bed and promotes the release of chemokines, thus promoting the recruitment and entry of activated immune cells to tumors (20, 21). These synergistic effects eventually lead to the invasion of CD8+ T cells and other effector cells into the TME, resulting in a strong local anti-tumor immune response (22). The release of immunomodulatory factors, activation of dendritic cells, and increased antigen presentation mediated by MHC I in tumor cells, induced by radiotherapy, can promote tumor-associated antigens’ immune recognition initiating anti-tumor T cells (23–25). Subsequently, tumor-associated antigen-specific cytotoxic T cells can attack tumor cells not only in irradiated local tumors but also in distant metastatic tumors (26).

Interestingly, this study showed that radiotherapy only significantly improved OS in mPCa patients with PSA levels of 4.1–10.0 ng/mL. Radiotherapy for mPCa with PSA < 4.0 ng/mL did not improve the survival rate. In the multivariate analysis, PSA was identified as an independent prognostic factor for mPCa, and the prognosis of the PSA in the < 4.0 ng/mL subgroup was lower than that of the PSA in the 4.1–10.0 ng/mL subgroup, which may explain why the curative effect of radiotherapy in the PSA < 4.0 ng/mL subgroup was not as good as that of the PSA in the 4.1–10.0 ng/mL subgroup. Wang et al. also pointed out that low PSA (< 4 ng/mL) is a unique entity that represents more invasive disease in mPCa and indicates a poor prognosis (12). It is reported that about 5–10% of PCa show low PSA (27); this may be due to the potential biological characteristics of dedifferentiation where epithelial cells lose the expression of PSA-coding genes (28), and patients with this type of PCa may have a higher incidence of non-organ-limiting diseases (29). In a study of 183 patients with mPCa, low PSA secretors’ molecular characteristics and clinical results were described. Compared with normal secretors, RB1 and TP53 gene deletions were more common in low PSA secretors. More importantly, similar to our results, patients with low PSA secretion had a shorter OS (30). Therefore, a low PSA level is an indicator of poorly differentiated aggressive tumor behavior, while a very high PSA level is an indicator of a high tumor burden (31). We believe that poor differentiation and a high tumor load may have a negative impact on the survival rate and efficacy of radiotherapy in patients with mPCa.

Boevé et al. provided important data for evaluating the quality of life in patients with mPCa after radiotherapy. In their recent study, they reported that patients who received radiotherapy had more diarrhea, urinary, and intestinal symptoms than those in the control group. Although the differences in urinary symptoms and diarrhea disappeared, the intestinal symptoms were still higher than those in the control group after two years (5). Therefore, the choice of radiotherapy for patients with mPCa should be carefully considered to avoid unnecessary complications.

There are some limitations to our study. Since it is a retrospective study, it is inevitably affected by some potential biases. Second, the database lacks detailed information on systemic treatments, androgen deprivation therapy, and radiation doses. The variables available in the SEER database may be inadequate to account for all the variation in treatment selection. Third, our data was obtained only from the SEER database since there are very few mPCa patients with low PSA levels in the clinic; consequently, there is no real-world data verification. Our results need to be verified by high-quality prospective randomized trials.

## Conclusions

In this assessment of a large, population-based dataset, we found that therapeutic effects in mPCa patients after radiotherapy varied depending on PSA level. This study shows that radiotherapy is most beneficial for mPCa patients with PSA levels of 4.1–10.0 ng/mL. We not only confirmed that radiotherapy provides no added benefit when the tumor load is high, but also proposed for the first time that radiotherapy for poorly differentiated mPCa patients does not significantly improve the prognosis. Our study provides direction to prospective clinical trials and clinical treatment in the future and is expected to change the existing treatment strategies for mPCa.

## Data Availability Statement

The original contributions presented in the study are included in the article/**Supplementary Material**. Further inquiries can be directed to the corresponding authors.

## Author Contributions

Study concept & design: ZT and ML. Data acquisition or data analysis/interpretation: LM, QZ, and YZ. Manuscript drafting or manuscript revision for important intellectual content: ZT and XinW. Approval of final version of submitted manuscript: ML and XuanW. Literature research: TM and MW. All authors contributed to the article and approved the submitted version.

## Funding

This study was financially supported by the Beijing Municipal Science and Technology Project (Z201100005620007), the Beijing Hospital Clinical Research 121 Project (BJ-2018-090), and the Fundamental Research Funds for the Central Universities (3332020069).

## Conflict of Interest

The authors declare that the research was conducted in the absence of any commercial or financial relationships that could be construed as a potential conflict of interest.
