# Supplementary material for: Survival in Patients With Metastatic Prostate Cancer Undergoing Radiotherapy: The Importance of Prostate-Specific Antigen-Based Stratification
Source: Front Oncol. 2021 Jun 10;11:706236. doi: 10.3389/fonc.2021.706236 (PMC8224529; doi:10.3389/fonc.2021.706236)
Supplement: Supplementary file 1 [file Table_1.docx]

Supplementary table 1 Univariate Cox regression analysis of treatment groups in PSA subgroup

| Group |  | OS univariate analysis |  |  | CSS univariate analysis |  |
| --- | --- | --- | --- | --- | --- | --- |
|  |  | HR (95%CI) | P value |  | HR (95%CI) | P value |
| **PSA ≤ 4** |  |  |  |  |  |  |
| Control group |  | Ref |  |  | Ref |  |
| Radiotherapy group |  | 0.850 (0.677-1.068) | 0.162 |  | 0.896 (0.691-1.161) | 0.405 |
| **PSA 4.1-10** |  |  |  |  |  |  |
| Control group |  | Ref |  |  | Ref |  |
| Radiotherapy group |  | 0.665 (0.580-0.762) | <0.001 |  | 0.684 (0.579-0.806) | <0.001 |
| **PSA 10.1-20** |  |  |  |  |  |  |
| Control group |  | Ref |  |  | Ref |  |
| Radiotherapy group |  | 0.904 (0.803-1.019) | 0.098 |  | 0.987 (0.858-1.137) | 0.860 |
| **PSA 20.1-40** |  |  |  |  |  |  |
| Control group |  | Ref |  |  | Ref |  |
| Radiotherapy group |  | 1.035 (0.928-1.154) | 0.540 |  | 1.091 (0.962-1.237) | 0.175 |
| **PSA 40.1-80** |  |  |  |  |  |  |
| Control group |  | Ref |  |  | Ref |  |
| Radiotherapy group |  | 1.024 (0.923-1.136) | 0.656 |  | 1.132 (1.007-1.273) | 0.038 |
| **PSA >80.1** |  |  |  |  |  |  |
| Control group |  | Ref |  |  | Ref |  |
| Radiotherapy group |  | 1.002 (0.955-1.051) | 0.941 |  | 1.086 (1.029-1.145) | 0.003 |

Abbreviations: HR, hazard ratio; 95%CI, 95% confidence intervals; OS，overall survival; CSS cancer specific survival
