# Supplementary material for: Survival in Patients With Metastatic Prostate Cancer Undergoing Radiotherapy: The Importance of Prostate-Specific Antigen-Based Stratification
Source: Front Oncol. 2021 Jun 10;11:706236. doi: 10.3389/fonc.2021.706236 (PMC8224529; doi:10.3389/fonc.2021.706236)
Supplement: Supplementary file 2 [file Table_2.docx]

Supplementary table 2 Multivariate Cox regression analysis of treatment groups in PSA 40.1-80 and >80.1ng/ml subgroups.

| Clinicopathological variables |  | CSS multivariate analysis (PSA 40.1-80ng/ml) |  |  | CSS multivariate analysis (PSA >80.1ng/ml) |  |
| --- | --- | --- | --- | --- | --- | --- |
|  |  | HR (95%CI) | P value |  | HR (95%CI) | P value |
| **Group** |  |  |  |  |  |  |
| Control group |  | Reference |  |  | Reference |  |
| Radiotherapy group |  | 1.122 (0.997-1.263) | 0.056 |  | 1.065 (1.009-1.124) | 0.023 |
| **Age at diagnosis** |  |  |  |  |  |  |
| ≤65 |  | Reference |  |  | Reference |  |
| >65 |  | 1.141 (1.022-1.275) | 0.019 |  | 1.247 (1.189-1.309) | <0.001 |
| **Race** |  |  |  |  |  |  |
| Caucasians |  | Reference |  |  | Reference |  |
| African Americans |  | 0.994 (0.859-1.150) | 0.939 |  | 1.019 (0.964-1.077) | 0.503 |
| Other/ Unknown |  | 0.700 (0.561-0.875) | 0.002 |  | 0.715 (0.646-0.791) | <0.001 |
| **T** |  |  |  |  |  |  |
| ≤T1 |  | Reference |  |  | Reference |  |
| T2 |  | 0.949 (0.828-1.088) | 0.456 |  | 1.071 (1.001-1.146) | 0.046 |
| T3 |  | 0.922 (0.764-1.112) | 0.395 |  | 0.994 (0.905-1.092) | 0.901 |
| T4 |  | 1.525 (1.291-1.802) | <0.001 |  | 1.232 (1.136-1.336) | <0.001 |
| Tx |  | 0.891 (0.737-1.076) | 0.231 |  | 1.143 (1.057-1.236) | 0.001 |
| **N** |  |  |  |  |  |  |
| N0 |  | Reference |  |  | Reference |  |
| N1 |  | 1.186 (1.041-1.351) | 0.011 |  | 0.976 (0.921-1.035) | 0.424 |
| Nx |  | 1.210 (1.058-1.384) | 0.005 |  | 1.088 (1.027-1.152) | 0.004 |
| **M** |  |  |  |  |  |  |
| M1a |  | Reference |  |  | Reference |  |
| M1b |  | 1.530 (1.200-1.951) | 0.001 |  | 1.791 (1.570-2.043) | <0.001 |
| M1c |  | 1.725 (1.328-2.241) | <0.001 |  | 2.121 (1.849-2.434) | <0.001 |
| M1x |  | 1.123 (0.762-1.656) | 0.556 |  | 1.822 (1.514-2.192) | <0.001 |
| **Gleason** |  |  |  |  |  |  |
| ≤6 |  | Reference |  |  | Reference |  |
| 7 |  | 0.883 (0.594-1.315) | 0.541 |  | 1.036 (0.849-1.264) | 0.727 |
| 8-10 |  | 1.421 (0.973-2.075) | 0.069 |  | 1.292 (1.069-1.562) | 0.008 |
| Unknown |  | 1.983 (1.329-2.958) | 0.001 |  | 1.498 (1.234-1.818) | <0.001 |

Abbreviations: HR, hazard ratio; 95%CI, 95% confidence intervals; OS，overall survival; CSS cancer specific survival
