# Supplementary material for: Survival in Patients With Metastatic Prostate Cancer Undergoing Radiotherapy: The Importance of Prostate-Specific Antigen-Based Stratification
Source: Front Oncol. 2021 Jun 10;11:706236. doi: 10.3389/fonc.2021.706236 (PMC8224529; doi:10.3389/fonc.2021.706236)
Supplement: Supplementary file 3 [file Table_3.docx]

Supplementary Table 3 Multivariate cox regression analysis of the prognostic factors for overall survival and cancer specific survival.

| Clinicopathological variables |  | OS multivariate analysis |  |  | CSS multivariate analysis |  |
| --- | --- | --- | --- | --- | --- | --- |
|  |  | HR (95%CI) | P value |  | HR (95%CI) | P value |
| **PSA** |  |  |  |  |  |  |
| <4 ng/ml |  | 1.331 (1.183-1.497) | <0.001 |  | 1.436 (1.252-1.647) | <0.001 |
| 4.1-10 ng/ml |  | Reference |  |  | Reference |  |
| 10.1-20 ng/ml |  | 1.136 (1.048-1.231) | 0.002 |  | 1.120 (1.017-1.235) | 0.021 |
| 20.1-40 ng/ml |  | 1.211 (1.121-1.309) | <0.001 |  | 1.257 (1.146-1.378) | <0.001 |
| 40.1-80 ng/ml |  | 1.379 (1.277-1.489) | <0.001 |  | 1.459 (1.332-1.597) | <0.001 |
| >80.1 ng/ml |  | 1.544 (1.444-1.651) | <0.001 |  | 1.679 (1.550-1.818) | <0.001 |
| **Group** |  |  |  |  |  |  |
| Control group |  | Reference |  |  | / |  |
| Radiotherapy group |  | 0.961 (0.926-0.997) | 0.033 |  | / | / |
| **Age at diagnosis** |  |  |  |  |  |  |
| ≤65 |  | Reference |  |  | Reference |  |
| >65 |  | 1.348 (1.303-1.394) | <0.001 |  | 1.184 (1.140-1.230) | <0.001 |
| **Race** |  |  |  |  |  |  |
| Caucasians |  | Reference |  |  | Reference |  |
| African Americans |  | 1.012 (0.971-1.053) | 0.580 |  | 0.990 (0.945-1.038) | 0.685 |
| Other/ Unknown |  | 0.718 (0.671-0.769) | <0.001 |  | 0.692 (0.639-0.749) | <0.001 |
| **T** |  |  |  |  |  |  |
| ≤T1 |  | Reference |  |  | Reference |  |
| T2 |  | 1.059 (1.014-1.105) | 0.009 |  | 1.057 (1.005-1.111) | 0.030 |
| T3 |  | 0.975 (0.916-1.037) | 0.412 |  | 1.009 (0.941-1.083) | 0.794 |
| T4 |  | 1.306 (1.237-1.379) | <0.001 |  | 1.361 (1.280-1.448) | <0.001 |
| Tx |  | 1.152 (1.090-1.217) | <0.001 |  | 1.160 (1.089-1.235) | <0.001 |
| **N** |  |  |  |  |  |  |
| N0 |  | Reference |  |  | Reference |  |
| N1 |  | 1.031 (0.989-1.074) | 0.148 |  | 1.070 (1.022-1.121) | 0.004 |
| Nx |  | 1.105 (1.062-1.150) | <0.001 |  | 1.117 (1.066-1.168) | <0.001 |
| **M** |  |  |  |  |  |  |
| M1a |  | Reference |  |  | Reference |  |
| M1b |  | 1.473 (1.364-1.591) | <0.001 |  | 1.688 (1.539-1.852) | <0.001 |
| M1c |  | 1.728 (1.593-1.875) | <0.001 |  | 1.983 (1.798-2.187) | <0.001 |
| M1x |  | 1.570 (1.399-1.761) | <0.001 |  | 1.732 (1.511-1.986) | <0.001 |
| **Gleason** |  |  |  |  |  |  |
| ≤6 |  | Reference |  |  | Reference |  |
| 7 |  | 1.181 (1.063-1.313) | 0.002 |  | 1.423 (1.238-1.636) | <0.001 |
| 8-10 |  | 1.638 (1.483-1.809) | <0.001 |  | 2.169 (1.900-2.476) | <0.001 |
| Unknown |  | 1.994 (1.794-2.216) | <0.001 |  | 2.544 (2.215-2.921) | <0.001 |

Abbreviations: HR, hazard ratio; 95%CI, 95% confidence intervals; OS，overall survival; CSS cancer specific survival
